# Isolation and purification of carbohydrate components in functional food: a review

**DOI:** 10.1039/d4ra02748e

**Published:** 2024-07-23

**Authors:** Chao Ji, Ying Ma, Yuxin Xie, Junli Guo, Haoran Ba, Zheng Zhou, Kongxiang Zhao, Min Yang, Xiahong He, Wenjie Zheng

**Affiliations:** a Tianjin Key Laboratory of Animal and Plant Resistance, College of Life Sciences, Tianjin Normal University Tianjin 300387 China skyzwj@tjnu.edu.cn; b The Animal, Plant & Foodstuff Inspection Center of Tianjin Customs Tianjin 300387 China; c State Key Laboratory for Conservation and Utilization of Bio-Resources in Yunnan, National Engineering Research Center for Applied Technology of Agricultural Biodiversity, College of Plant Protection, Yunnan Agricultural University Kunming Yunnan 650201 China hexiahong@hotmail.com; d Key Laboratory for Forest Resources Conservation and Utilization in the Southwest Mountains of China, Ministry of Education, Southwest Landscape Architecture Engineering Research Center of National Forestry and Grassland Administration, Southwest Forestry University Kunming Yunnan 650224 China

## Abstract

Medicinal plants, increasingly utilized in functional foods, possess potent therapeutic properties and health-promoting functions, with carbohydrates playing a crucial role and exhibiting a range of effects, such as antioxidant, antitumor, immune-enhancing, antibacterial, anticoagulant, and hypoglycemic activities. However, comprehensively, accurately, rapidly, and economically assessing the quality of carbohydrate components is challenging due to their diverse and complex nature. Additionally, the purification and identification of carbohydrates also guarantee related efficacy research. This paper offers a thorough review of research progress carried out by both domestic and international scholars in the last decade on extracting, purifying, separating, identifying, and determining the content of carbohydrate components from functional foods, which are mainly composed of medicinal plants, and also explores the potential for achieving comprehensive quantitative analysis and evaluating structure–activity relationships of carbohydrate components. These findings aim to serve as a valuable reference for the future development and application of natural carbohydrate components in functional food and medicine.

## Abstract 2

The workflow for carbohydrate extraction, separation and detection steps.

## Graphical abstract

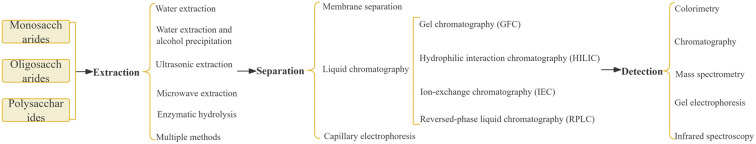

## 1. Introduction

**Fig. 1:**
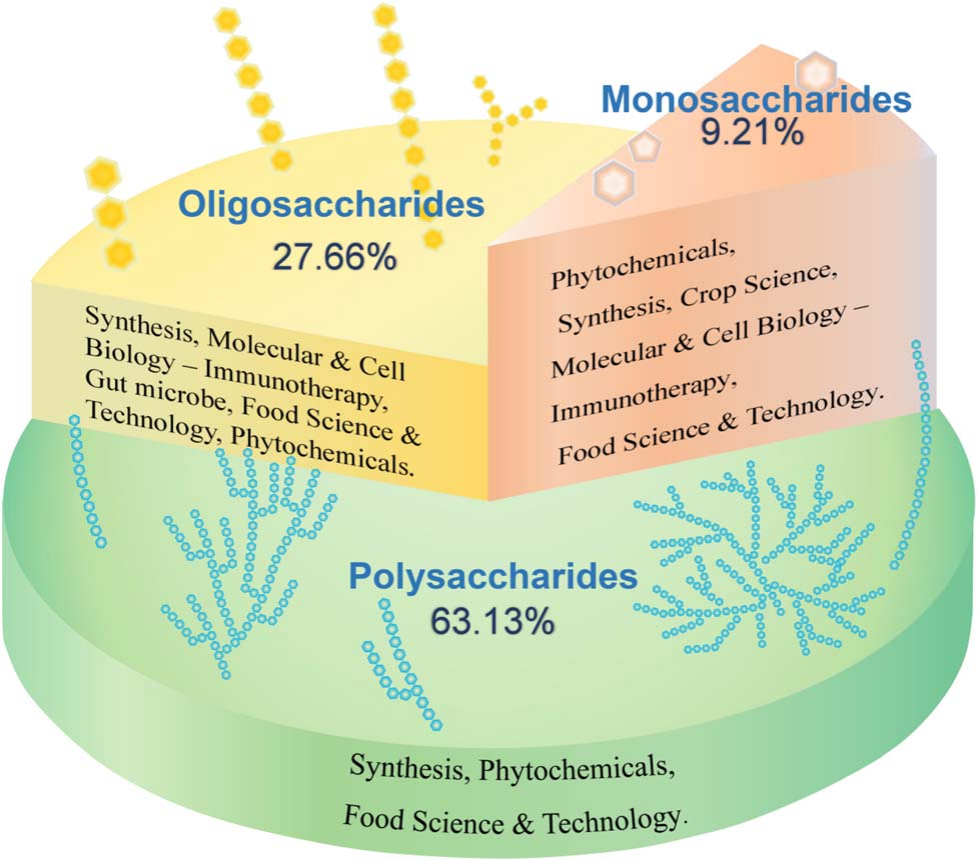
Research status of various carbohydrate components.

Carbohydrates constitute a class of organic compounds abundant in nature, categorized into monosaccharides, oligosaccharides, and polysaccharides based on their degree of polymerization.^1^ They are widely distributed in various organisms, including the seeds, stems, and leaves of herbaceous plants, animal body fluids, cell walls, and extracellular fluids of bacteria, yeasts, and fungi.^2^ The intricate structure and diverse functions of carbohydrates have led to a significant increase in research over the past five years, primarily in the fields of phytochemicals and food science and technology. A search of the Web of Science database using the keywords “monosaccharides”, “oligosaccharides”, and “polysaccharides” revealed that oligosaccharides have the largest number of studies (50 718 articles, accounting for 27.66%), primarily focusing on their synthesis pathway, therapeutic function (immunity) at molecular and cellular levels, intestinal microorganisms, food science, phytochemistry and other fields. Research on monosaccharides (16 879 articles, accounting for 9.21%) has primarily focused on phytochemistry, with studies in synthesis, crop science, molecular and cell biology, food science, and other fields. Most studies have focused on polysaccharides (115 761 articles, accounting for 63.13%), primarily in the field of phytochemistry, with a focus on the overall carbohydrate components (Fig. 1). However, the diversity of structure–activity relationships formed by different types of monosaccharide units and glycosidic bond connections in the macromolecular polysaccharides of carbohydrate components has received limited attention.

Researchers worldwide have long investigated the medicinal and food properties of medicinal plants, and these plants have a rich historical background and have made significant contributions to human civilization.^3,4^ The application of bioactive saccharides from medicinal plants has garnered significant attention in the fields of medicine, biomedicine, and functional foods. Kiyohara *et al.*^5^ isolated 13 different types of polysaccharides in *Astragalus membranaceus*, nine of which were composed of arabinogalactan and pectic acid. *Astragalus* polysaccharides have been found to improve immune cell function and exhibit strong immunomodulatory effects. Ginseng polysaccharides include arabinogalactan, pectin, and acidic polysaccharides. These polysaccharides are primarily composed of monosaccharides such as l-arabinose, d-galactose, l-rhamnose, d-galacturonic acid, and d-glucuronic acid. Ginseng polysaccharides possess a range of biological properties, including antibacterial, antioxidant, anti-inflammatory, antidepressant, antitumor, and immunomodulatory effects both *in vitro* and *in vivo*.^6,7^*Angelica* polysaccharides are rich in galacturonic acid, galactose, and arabinose^8^ and have been found to exhibit antitumor, antioxidant, and intestinal barrier protection functions.^9–12^*Polygonatum* polysaccharides are the primary active ingredients of *Polygonatum sibiricum*;^13^ they are mainly neutral fructans,^14^ exhibiting antioxidant,^15–18^ anti-aging,^19^ and immune-regulatory^20–25^ properties. *Glycyrrhiza* polysaccharides exhibit diverse biological activities, including antioxidant, immune-regulating, antitumor, cell apoptosis, antibacterial, anti-inflammatory, and intestinal flora regulation.^26^ Carbohydrates serve a crucial role as the primary active components in most medicinal plants, exhibiting diverse functions and beneficial effects. However, in the previous research on the detection method of carbohydrate components, the process of extraction and purification took a long time, and most of them were extracted from the crude saccharides solution, and then the carbohydrate components were separated by various separation methods. The steps are cumbersome, which is not conducive to rapid overall analysis of various carbohydrate components; in terms of content determination, at present, scholars at home and abroad have done more research on the determination methods of monosaccharide and oligosaccharide components, while polysaccharides are complex and diverse due to the influence of connecting sites, saccharides chain structure and branching, and there are few studies on polysaccharide components. Efficient and cost-effective quantitative analysis of carbohydrate components in medicinal plants is a significant research focus.

**Fig. 2:**
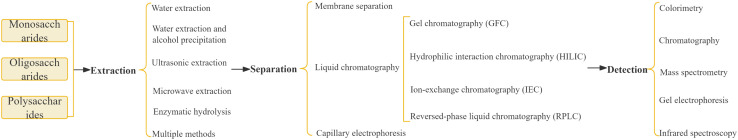
The workflow for carbohydrate extraction, separation and detection steps.

Therefore, this paper provides a comprehensive review of recent research on the extraction, purification, separation, identification, and content determination of carbohydrate components in medicinal plants. The aim of this review is to explore efficient and cost-effective methods for extracting and separating various carbohydrate components from medicinal plants, as well as to quantitatively detect them with high efficiency (Fig. 2). The findings of this review can serve as technical guidance for quality control of carbohydrate components in medicinal plants and provide strong support for the future development and application of natural carbohydrate components in functional food and medicine.

## 2. Classification and functions of carbohydrate components

Monosaccharides such as glucose, galactose, ribose, and deoxyribose are fundamental units of carbohydrates. The type and proportion of monosaccharides in polysaccharides are closely linked to their biological activity, with greater complexity in monosaccharide composition generally leading to enhanced biological activity.^27^ For example, *Angelica* polysaccharides have radioprotective activity because of their high galacturonic acid, galactose, and arabinose contents.^8^ Polysaccharides with intestinal barrier protection functions mostly contain galactose, mannose, arabinose, xylose, and rhamnose as monosaccharides.^9,10^ Polysaccharides with high uronic acid content exhibit better antioxidant activity,^28^ possibly through the breakage of the uronic acid chain caused by free radicals.^29^ Polysaccharides containing a certain amount of uronic acid exhibit hepatoprotective activities.^27^ Polysaccharides with mannose and rhamnose exhibit antitumor and antioxidant activities, respectively.^11,12^ Additionally, the types of glycosidic bonds and the degree of branching also affect carbohydrate function. For example, the biological activity of α-d-(1→3) glucan is restricted by its hydrophobicity.^30^

Oligosaccharides are an important class of carbohydrates consisting of 2-10 identical or different monosaccharides connected by glycosidic bonds, forming either straight or branched chains. The main constituent units are five- or six-carbon saccharides, with glucose, fructose, galactose, xylose, arabinose, and mannose being the most prevalent. These compounds commonly form covalent bonds with proteins or lipids, existing as glycoproteins or glycolipids, and have been found to possess various pharmacological and physiological functions, including blood sugar reduction,^31^ anti-post-traumatic stress disorder,^32^ antitumor,^33^ antibacterial,^34^ intestinal flora-regulating,^35^ and anti-depression effects.^36^

**Fig. 3:**
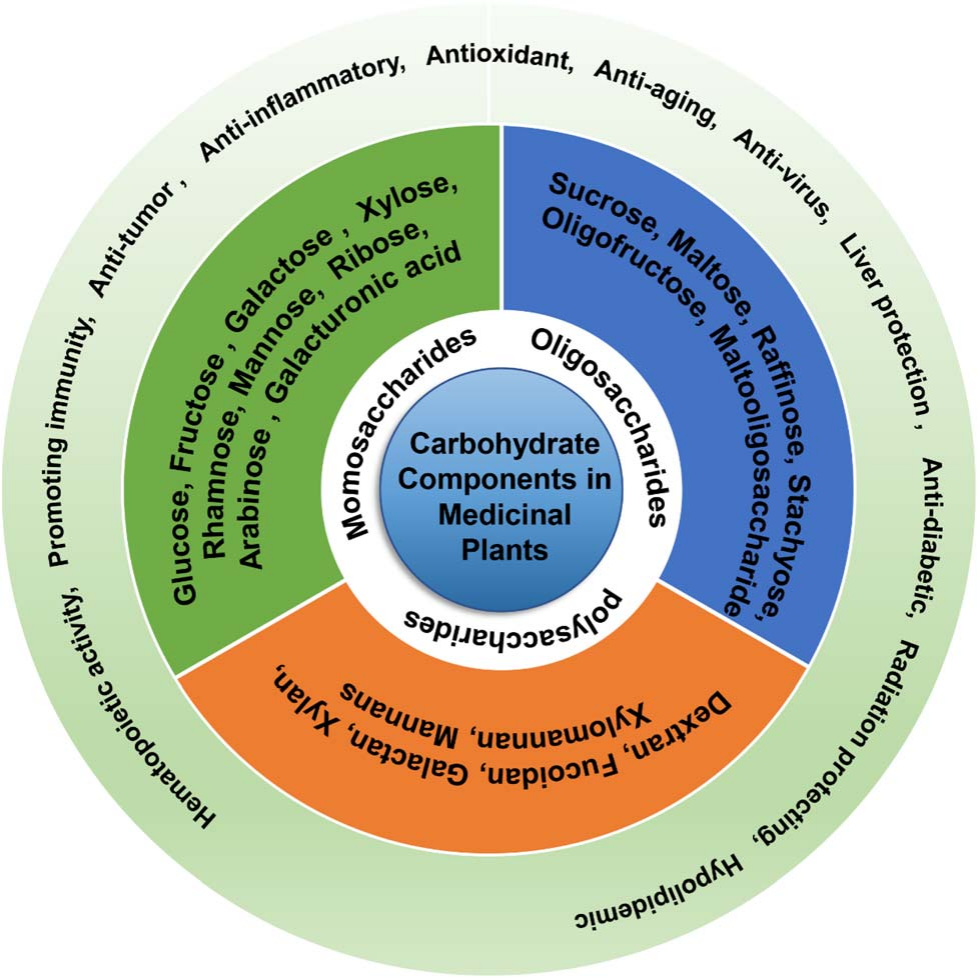
Function and classification of carbohydrate components in medicinal plants.

Polysaccharides can be categorized into homopolysaccharides, heteropolysaccharides, acidic polysaccharides, neutral polysaccharides, basic polysaccharides, storage polysaccharides, and structural polysaccharides based on their function, shape, and chemical properties. Examples of polysaccharides include dextran, fucoidan, galactan, xylan, xylomannan, and mannan.^14^ Recent pharmacological studies have shown that medicinal plant-derived polysaccharides possess antitumor,^37^ immune-enhancing,^38^ intestinal microenvironment-regulating,^39^ antioxidant,^40^ anti-coagulating,^41^ and anti-diabetic^42^ properties. Prominent examples of medicinal plants that contain polysaccharides with diverse structures and biological activities and possess diverse pharmacological activities include *Astragalus*,^43^ ginseng,^44^*Angelica*,^45^*Polygonatum*,^46^ and Licorice.^26^ The chemical characteristics and biological activities of polysaccharides from these plants have been extensively studied and reported. The function and classification of the carbohydrate components in medicinal plants are shown in Fig. 3.

## 3. Extraction and separation of carbohydrate components

### 3.1 Extraction

Carbohydrate components in medicinal plants are typically extracted using various methods, including water extraction, water extraction and alcohol precipitation,^13,47,48^ ultrasonic extraction,^49,50^ microwave extraction,^51,52^ enzymatic hydrolysis,^53^ or a combination of multiple methods.^51,52,54^

Water extraction, as a traditional carbohydrate component extraction method, mainly uses hot water extraction to dissolve polar macromolecular compound polysaccharides in water and other polar solvents, and uses the principle of “Substances with similar properties are compatible” for extraction. In addition, according to the structure and properties of polysaccharides, with the help of some auxiliary means, on the basis of traditional water extraction, acid–base extraction, enzyme extraction, microwave-assisted extraction, ultrasonic-assisted extraction and ultra-high-pressure extraction have been developed.^54^ As the most widely used carbohydrate component extraction method, water extraction and alcohol precipitation method is suitable for the extraction of various components such as monosaccharides, oligosaccharides and polysaccharides. Its principle is to use the characteristics that monosaccharides and oligosaccharides in sugar components are soluble in water but not in ethanol, while polysaccharides are soluble in alcohol, so as to reduce the solubility of polysaccharides, precipitate and achieve solid–liquid separation. Li *et al.*^48^ obtained *Platycodon grandiflorus* polysaccharides by combining hot water extraction with ethanol precipitation, that is, water extraction and alcohol precipitation method; fructooligosaccharides in *Polygonatum* can also be extracted by water extraction and alcohol precipitation.^47^ Ultrasonic-assisted extraction based on the principle of water extraction and alcohol precipitation is more efficient than the traditional water extraction and alcohol precipitation method. Zhao *et al.*^50^ adopted ultrasonic-assisted extraction of corn silk polysaccharides, and optimized it by response surface methodology. The results showed that the yield of corn silk polysaccharide obtained by ultrasonic optimized extraction (yield 7.31%) was higher than hot water extraction (yield 5.46%) and microwave-assisted extraction (yield 6.18%).^49^ Other studies have shown that the extraction effect is more remarkable by combining ultrasonic extraction with microwave extraction. Huang *et al.*^51^ used ultrasonic and microwave-assisted extraction (UMAE) to extract polysaccharides from Ganoderma lucidum, and the yield of polysaccharide was 115.56% higher than that obtained by traditional hot water extraction, and 27.7% higher than that obtained by ultrasonic-assisted extraction. Shen *et al.*^52^ used ultrasonic/microwave-assisted extraction (UMAE) to extract *Panax notoginseng* polysaccharide (PNPS), and optimized the ultrasonic time, ultrasonic power, microwave time and microwave power by using the response surface method, which improved the extraction efficiency, shortened the processing time, and reduced the solvent consumption and required energy. At present, there are also studies on introducing two enzymes, α-amylase and cellulase, into the extraction system to destroy granular starch and plant cell walls through enzymatic hydrolysis, so as to effectively extract sugar components with different structures and activities.^53^

This paper presents a systematic comparison and analysis of the advantages and disadvantages of various extraction methods, which are summarized in Table 1. Compared to traditional methods, the use of multiple instruments in the extraction method improves efficiency and reduces processing time. Traditional water extraction has low efficiency, whereas alcohol precipitation and reflux extraction have significantly improved extraction effects, but require a considerable amount of extraction solvent and are time-consuming. Ultrasonic extraction, microwave extraction, and enzymatic hydrolysis can expedite polysaccharide hydrolysis into various components including monosaccharides and oligosaccharides, which are typically extracted using water extraction methods. To precipitate polysaccharides from the TCM extract, ethanol is added to the water extraction process owing to the presence of monosaccharides, oligosaccharides, and proteins. In addition, oligosaccharides are relatively stable in solvents with ethanol volume fractions of 40% or above^55^ and exhibit limited susceptibility to hydrolysis and formation of unknown components.

**Table 1:** Comparison of the main extraction methods for carbohydrate components in medicinal plants

Methods	Scope of application	Advantages	Shortcomings	References
Water extraction and alcohol precipitation	Pretreatment of each polysaccharide component. It is suitable for use as the basis of extraction in conjunction with other extraction methods to improve extraction efficiency	Wide range of applications and complete dissolution	Large solvent consumption and time-consuming	20, 47 and 50
Ultrasonic extraction	Can significantly break the polysaccharide structure in a short time and is suitable for extracting polysaccharides with high efficiency	More extraction components, high efficiency and less reagent consumption	Poor repeatability	49 and 51
Microwave extraction	Suitable for extracting polysaccharides with high efficiency	Strong penetration power, high selectivity, high extraction rate, and good reproducibility	Restricted extraction solvent	52 and 53
Enzymatic method	Pretreatment for detecting polysaccharide components	Strong specificity and high catalytic rates	Easy inactivation and small application range	54
Ultrasound microwave-assisted extraction	Extraction of crude polysaccharide components, suitable for quick and efficient extraction of polysaccharides	High efficiency and less solvent requirement	Complex operation	52 and 53

Methods for extracting carbohydrates are transitioning from the traditional high material-liquid ratio, time-consuming, complicated operations with low efficiency to low material-liquid ratio, shorter extraction times, simpler operations, and a combination of multiple extraction techniques, these methods offer improved extraction efficiency and purity. When researching and developing new extraction methods, it is crucial to prioritize the yield of high-quality oligosaccharides. This can be achieved by combining the advantages of the established techniques with the latest equipment and methods, with the goal of developing an eco-friendly, cost-effective, and efficient extraction method.

### 3.2 Separation

Carbohydrate components in medicinal plants are typically isolated using membrane separation,^56–58^ liquid chromatography,^59–63^ and capillary electrophoresis.^64^

#### 3.2.1 Membrane separation technology

Membrane separation technology involves selective separation of molecules with varying particle sizes at the molecular level using semi-permeable membranes. Mechanical sieving is employed in this technique to selectively separate the target substance from the solution. The widespread popularity of applying membrane separation technology in TCM is attributed to its high efficiency, low energy consumption, and lack of pollution. In terms of separation and purification of oligosaccharides, Cai *et al.*^56^ established an integrated membrane separation system using microfiltration, ultrafiltration, and nanofiltration membranes, and used this system to separate crude oligosaccharides from *Hericium erinaceus* with a content of 14.84% and a purity of 63.71%. Li *et al.*^57^ used nanofiltration membranes NF-3A and NF-2A to construct a constant volume diafiltration method to separate and purify soybean oligosaccharide fermentation broth, with a yield of 3.2% and a purity of 77.9%. In the process of oligosaccharide separation using membrane filtration, it is crucial to select a filter membrane with an appropriate pore size based on the molecular size of the target isolate. Coarse filtration methods, like microfiltration with large pore sizes, lack accuracy. Ultrafiltration is commonly employed for the removal of proteins or macromolecular components,^58^ while nanofiltration membranes are effective in eliminating small molecular sugars.^57^ The use of membrane separation methods results in higher quantities and purer oligosaccharides. However, this method is unsuitable for separating oligosaccharides with similar molecular weights. The application of this technology necessitates high-quality equipment and membranes, involves a complex operation, and is affected by various factors, including pressure, temperature, and time.

#### 3.2.2 Liquid chromatography (LC)

Liquid chromatography (LC) is the most widely used technique for the separation and analysis of monosaccharides and oligosaccharides. This technique can be classified into gel exclusion chromatography, hydrophilic interaction chromatography,^59,61^ anion exchange chromatography,^65^ graphitized carbon column chromatography, and reversed-phase high-performance liquid chromatography.^60^

#### 3.2.3 Gel chromatography (GC)

Gel chromatography (GC) achieves compound separation by utilizing their molecular size. Smaller molecules can enter the pores of the material, resulting in a longer retention time before elution, whereas larger molecules have shorter retention times. Depending on the type of mobile phase used,.GC can be categorized into gel filtration chromatography (GFC) and gel permeation chromatography (GPC). A study used ultrasonic-assisted extraction to extract seed polysaccharides (PCSP) from *Pouteria campechiana*, and separated and purified them through cellulose column and Sephadex column to obtain PCSPa-1 pure polysaccharide fraction;^63^ Liu *et al.*^62^ extracted water-soluble crude polysaccharide (PS50) from *Polygonatum sibiricum*, dialyzed after deproteinization, PS50 was separated and purified by DEAE-52 cellulose and Sephadex G-75 gel filtration chromatography, and finally obtained two new polysaccharides (PSP50-2-1 and PSP50-2-2).

#### 3.2.4 Hydrophilic interaction chromatography (HILIC)

Hydrophilic interaction chromatography (HILIC) depends on the hydrophilic interactions between the material's surface functional groups and saccharide chains. The high hydrophilicity of saccharide chains is attributed to the numerous hydroxyl groups present in monosaccharides. Chemically bonded diol groups, amino groups, saccharide groups, cyclodextrins, polyethylene glycol, alkyl groups in amides or carbamates, and polymer-bonded phases^59^ are commonly used as stationary phases for carbohydrate separation. Hao *et al.*^61^ separated and determined the extract of *Morinda officinalis* using an amide-bonded chromatographic column, and successfully quantitatively analyzed 13 kinds of carbohydrate components in *Morinda officinalis*, of which the content of 10 kinds of inulin oligosaccharides was 56.28–60.71%.

#### 3.2.5 Ion-exchange chromatography (IEC)

Ion-exchange chromatography (IEC) is a liquid chromatographic separation technique utilizing an ion exchanger as a stationary phase to separate components based on their differing ion-exchange capacities. Ionic groups on the stationary phase in IEC can interact with specific groups of the separated components. During elution with the mobile phase, carbohydrate components are eluted based on their binding abilities, with smaller components eluting first followed by larger ones, ensuring effective separation. IEC offers advantages such as reduced sample consumption, high separation efficiency, and high sensitivity. It is suitable for the separation of acidic carbohydrate components in high-pH mobile phases. Nevertheless, its qualitative ability is limited, and it can be combined with other chromatography methods to improve separation and purification. Le *et al.*^65^ used ion chromatography and pulsed amperometric detector to simply, quickly and accurately determine the content and composition of seven monosaccharides in aloe polysaccharides.

#### 3.2.6 Reversed-phase liquid chromatography (RPLC)

Reversed-phase liquid chromatography (RPLC) typically employs binary or ternary mixed solutions, mainly consisting of acetonitrile, methanol, and water, as mobile phases for saccharide compound analysis. The ratio can be adjusted based on experimental requirements. If the initial conditions are unsatisfactory, gradient elution can enhance separation and reduce elution time. Moreover, the elution order of monosaccharides and oligosaccharides is correlated to their molecular weights. Fan *et al.*^60^ separated and quantitatively analyzed *Osmanthus fragrans* polysaccharides by pre-column derivatization HPLC-MS/MS and electrospray ionization (ESI); and effectively separated and quantitatively analyzed six monosaccharide components in three *Osmanthus fragrans* by reversed-phase liquid chromatography.

#### 3.2.7 Capillary electrophoresis (CE)

Capillary electrophoresis (CE) is an exceptionally efficient separation technique utilizing a capillary as the separation channel and a high-voltage direct current electric field as the driving force. CE is commonly employed in the analysis of saccharides, and offers advantages in terms of speed, efficiency, sensitivity, and separation efficiency, along with reduced sample and solvent consumption. Nevertheless, its implementation demands stringent equipment and operating conditions, leading to low separation efficiency. Ma *et al.*^64^ separated the monosaccharides in saffron corm glycoconjugates by CE combined with pre-column derivatization, and achieved baseline separation of 11 monosaccharides and disaccharides.

Table 2 provides a summary of the advantages and disadvantages of different methods for separating carbohydrate components. Membrane separation demonstrates high efficiency, low energy consumption, and no pollution. This enables molecular-level filtration, facilitating efficient and controllable separation, concentration, purification, and refining. While membrane separation provides distinct advantages in purification and impurity removal, it demands high equipment and membrane standards, involves complex operations, and is sensitive to various factors, including pressure, temperature, and time. Membrane separation is primarily employed for the separation of oligosaccharides with high yield and purity; however, it is unsuitable for separating oligosaccharides with similar molecular weights. Column chromatography is extensively utilized for the separation and purification of polysaccharides. The choice of separation materials should be guided by the properties of monosaccharides and oligosaccharides to achieve optimal separation and purification. Currently, the research focus, especially in the natural products field, is on enhancing separation materials for saccharide compounds. Oligosaccharide separation through electrophoresis encompasses four methods: pre-column derivatization, direct/indirect UV detection (without derivatization), laser-induced fluorescence detection, and electrode pulse amperometric detection. Indirect UV detection can identify non-reducing oligosaccharides and aldonic acids that are challenging to derivatize, rendering them suitable for drug analysis and with the potential for further development. Nevertheless, challenges such as sensitivity, repeatability, and qualitative ability require improvement and breakthroughs.

**Table 2:** Comparison of separation methods for carbohydrate components in medicinal plants

Methods	Scope of application	Sample	Advantages	Shortcomings	References
Membrane separation method	Oligosaccharides with different molecular weights	Soybean/almond shell	Simple operation	Easy to block, and difficult to distinguish between carbohydrates with similar molecular weights	57 and 58
Gel chromatography	Monosaccharides and oligosaccharides of different molecular weights	The rhizome of *Polygonatum sibiricum*/*Pouteria campechiana* seed	Simple operation	Not suitable for industrial production	62 and 63
Hydrophilic interaction chromatography	Polar and hydrophilic compounds	—/*Morinda officianalis*	Good repeatability	Limited by sample volume	59 and 61
Ion exchange chromatography	Neutral and acidic carbohydrate components	Aloe	High sensitivity, simple operation, and high specificity	Unstable separation conditions	65
Reversed-phase liquid chromatography	Non-polar, polar, or ionic compounds	*Osmanthus fragrans*	High selectivity and good reproducibility	Not suitable for polar and hydrophilic compounds	60
Capillary electrophoresis	Samples with fluorescence reaction and charge	Saffron corm	Simple operation, high sensitivity, good separation, and good reproducibility	High equipment requirements	64

## 4. Carbohydrate components detection methods

Common methods for the qualitative and quantitative detection of sugar components in medicinal plants include colorimetry,^66^ chromatography,^67–75^ mass spectrometry,^48,76–79^ electrophoresis^46,80–83^ and their combinations.^84^

### 4.1 Colorimetry

Colorimetry is a commonly used technique for analyzing the total polysaccharide, starch, and pectin contents of medicinal plants. Phenol- and anthrone-sulfuric acid methods are commonly used to quantify the total polysaccharides in TCMs. However, colorimetric methods cannot provide detailed information regarding the bond positions and branches of polysaccharides. Consequently, these methods are not suitable for identifying and separating the different carbohydrate components in medicinal plants.^66^

### 4.2 Chromatography

High-performance liquid chromatography (HPLC) is commonly used for the detection of carbohydrate components in medicinal plants. However, analysis of polysaccharide components is challenging because of the complexity of their saccharide chains, and this method is limited to monosaccharide and oligosaccharide components. Gas chromatography (GC) is the primary approach for identifying the glycoside chain connections of oligosaccharides and polysaccharides.^73^ HPLC is an analytical technique for efficient separation, and the selection of its detector is crucial for accurate analysis of carbohydrate components. Commonly used detectors include ultraviolet (UV), refractive index (RI), and evaporative light-scattering (ELSD) detectors. The RI method is the conventional detection method following HPLC separation,^68^ but it can be affected by factors such as analyte elution, temperature, and eluent composition, leading to poor sensitivity of the RI method.^70^ Before detection by a UV detector, carbohydrate components must undergo derivatization with chemical reagents to introduce detectable groups for UV detection, followed by quantitative analysis.^69,71^ 1-Phenyl-3-methyl-5-pyrazolone (PMP) is commonly used as a derivatization reagent owing to its quantitative reaction with carbohydrate components.^67^ ELSD quantitatively detects the target substance by measuring the particles formed from the evaporated eluent of the analyte, with high sensitivity and without altering the saccharide structure. However, its calculation relationship is more complicated than those of the other two methods.

High-performance thin-layer chromatography (HPTLC) is widely employed for detecting carbohydrate component, benefiting from its color reaction, ease of operation, and straightforward result interpretation.^72,74^ Nonetheless, qualitative analysis using reference substances is required in HETLC, and it lacks the ability to quantify detected components.

### 4.3 Mass spectrometry

Mass spectrometry (MS) is widely used for analyzing the structure of carbohydrate components, with GC-MS being initially used for the analysis of saccharide composition and linkage patterns. Advancements in ionization technology have facilitated the development of soft ionization techniques, including electron bombardment mass spectrometry (EI-MS), fast atom bombardment mass spectrometry (FAB-MS), matrix-assisted laser desorption-time-of-flight mass spectrometry (MALDI-TOF MS), and electrospray ionization-mass spectrometry (ESI-MS), which have significantly enhanced the research on the molecular weight and connection order of sugar residues. The combination of these techniques has also improved the speed and efficiency of qualitative and quantitative analyses of monosaccharides and oligosaccharides. ESI-MS is a particularly important technique for analyzing oligosaccharide structures because of its high sensitivity, accuracy, and rapid characterization. Hu *et al.*^77^ used MS/MS method to determine six kinds of monosaccharides produced by *Polygonatum sibiricum* (PCH), and used MRM scanning method to quantitatively and qualitatively detect the monosaccharide components in the polysaccharide. The results showed that the polysaccharide contained glucose, mannose, rhamnose, galactose, ribose and arabinose. Xu *et al.*^79^ established a specific ultra-high performance liquid chromatography quadrupole trap tandem mass spectrometry (UHPLC-QTRAP-MS/MS) method for the determination of the monosaccharide composition of *Lycium barbarum* polysaccharide (LBP), and the results showed that, LBP is mainly composed of seven kinds of monosaccharides: galactose, arabinose, mannose, rhamnose, xylose, ribose, and glucose. Bai *et al.*^76^ used gas chromatography-mass spectrometry to analyze the structure of *Codonopsis* oligosaccharides obtained by water extraction and alcohol precipitation. The results showed that the main monosaccharides of *Codonopsis* oligosaccharides were fructose and glucose (α-Glcp, Glcp, β-Fruf, β-Fru), with a molar ratio of 1.21 : 1, the oligosaccharides are analyzed by GC-MS after methylation and derivatization, and the glycosidic linkage is →1-α-d-Glcp,→ 2) -β-d-Fruf-1(→ and β-d-Fruf-(2→. Lu *et al.*^78^ developed a method based on liquid chromatography-tandem mass spectrometry (LC-MS/MS) to completely characterize the monosaccharide composition in the *Ganoderma lucidum* polysaccharide GLP.

### 4.4 Gel electrophoresis

Although the complete structural elucidation of carbohydrate components enables the accurate characterization and identification of isolated polysaccharides^80^ and released *N*-glycans,^83^ these methods are typically impractical for routine quality analysis of water-extracted liquids containing diverse carbohydrates. Researchers have developed sugar maps using endoglycosidase digestion techniques, such as high-performance thin-layer chromatography (HPTLC), polysaccharide analysis using carbohydrate (glycan) gel electrophoresis (PACE), and high-performance size-exclusion chromatography (HPSEC). These methods are used to identify and analyze the quality of medicinal plant polysaccharides. They provide high repeatability, stability, sensitivity, and throughput.^82^ Saccharide detection based on PACE has proven to be an effective technique for the routine analysis of oligosaccharides.^81^ Chen *et al.*^46^ compared acid hydrolysates and hydrolysates of *Polygonatum* polysaccharide using PACE analysis to assess their carbohydrate composition. Developing a simple, fast, accurate, and specific qualitative and quantitative method for the quality control of carbohydrate components in medicinal plants has always been a challenge owing to their complexity. Polysaccharide map analysis using PACE has the characteristics of simplicity, good repeatability, high resolution, and high throughput, and has been proven to be one of the most effective methods for the quality control of carbohydrate components in natural resources.

### 4.5 Infrared (IR) spectroscopy

IR spectroscopy is primarily used to analyze the structure of oligosaccharides and polysaccharides, with a particular focus on determining the functional groups and glycosidic bond configurations. The primary characteristic peaks of the IR spectrum of the oligosaccharides include a prominent broad peak at 3300 cm^−1^, corresponding to the stretching vibration of –OH, and a weaker stretching band at approximately 2936 cm^−1^, corresponding to the stretching vibration of the –CH single bond. The absorption peak at 1740 cm^−1^ signifies the presence of uronic acid, whereas the absorption peaks at 1640 cm^−1^ and 1100 cm^−1^ represent the stretching vibrations of C=O and CO, respectively. The absorption at approximately 1400 cm^−1^ represents the bending vibration of –CH. The strong absorption in the 1200–1000 cm^−1^ range indicates the presence of COC glycosidic bonds as well as the stretching vibrations of the CC and C–OH bonds. Additionally, the absorption peak at 1024 cm^−1^ corresponds to the pyranose unit of oligosaccharides, whereas the absorption peaks at 930 cm^−1^ and 820 cm^−1^ suggest the presence of β- and α-glycosidic furanose rings.^47^

Table 3 summarizes the advantages and disadvantages of various carbohydrate component detection methods. Monosaccharides and oligosaccharides are typically separated and detected using UV and thin-layer chromatography for qualitative analysis of fluorescent or color-developing saccharides. LC is used for the separation and quantification of monosaccharides and oligosaccharides. IR absorption spectroscopy is generally used for the preliminary characterization of oligosaccharide structures by identifying the saccharide ring skeletons and functional groups. MS is generally used to determine polysaccharide saccharide chains and molecular weights, and plays a significant role in the structural identification of oligosaccharides and polysaccharides. Carbohydrate component detection can be enhanced by combining multiple methods tailored to each component based on specific situations owing to the limitations associated with different detection methods.

**Table 3:** Comparison of detection and analysis methods for carbohydrate components in medicinal plants

Methods	Scope of application	Advantages	Shortcomings	References
Colorimetry	Determination of the total polysaccharide content	Easy operation and intuitive results	Different polysaccharide cannot be identified or separated	66
HPLC	Monosaccharides and oligosaccharides of different molecular weights	High sensitivity and accuracy, effectively quantify various polysaccharide	Difficult to obtain a complex structure and content of polysaccharides	67–75
HPTLC	Color reaction	Easy operation and intuitive results	Required qualitative reference substance	72 and 74
Mass spectrometry	Analysis of polysaccharide component structure	High sensitivity, high precision, and fast characterization	High equipment requirements	47, 76–79
Gel electrophoresis	Various polysaccharide components after hydrolysis	High repeatability, stability, sensitivity, low cost, high output	Required reference substance but cannot be quantified	16, 80–83
Infrared spectroscopy	Determination of the functional groups	Simple, fast, environment-friendly, and widely used	Low sensitivity	50

## 5. Conclusion and outlook

Current research on carbohydrate components is in the preliminary stage and has several limitations. The complexity and diversity of saccharide chains in carbohydrate components have specific requirements for detection methods, such as the nature, purity, or dosage of the target saccharide. To address this, future development trends involve the combined application of multiple methods, such as water extraction and alcohol precipitation, combined with techniques such as ultrasonic, microwave, and enzymatic hydrolysis. Another approach is the use of ultrasonic microwave-assisted extraction^52^ to refine the extraction conditions and effectively separate the components of monosaccharides, oligosaccharides, and polysaccharides. Additionally, the combination of various detection methods enables the comprehensive and high-throughput detection of carbohydrate components in medicinal plants. Advancements in extraction methods, separation techniques, and detection and characterization methods for carbohydrate components enable efficient preparation, rapid separation, and accurate characterization and quantification of these components. This progress has enhanced the significance of carbohydrate components in medicinal plants for applications in food, medicine, and other fields.

Research on carbohydrates has experienced significant growth in recent years, particularly in the fields of pharmacology and health functions. Currently, research on carbohydrates primarily focuses on their activities and disease resistance mechanisms, with few studies exploring the structure–activity relationship and absorption metabolism of carbohydrates. The study of the carbohydrate components in medicinal plants should focus on different glycosidic linkages, as they exhibit distinct biological activities. Monosaccharides are linked to form polysaccharides that possess complex structures. The glycosidic bond can be classified as α- and β-type, depending on the configuration of the hemiacetal (ketone) hydroxyl group. β-Configuration are generally more active than α-configuration polysaccharides.^85,86^ The presence of α-glucoamylase in the human body facilitates the hydrolysis of α-glycosidic bonds under specific conditions. Recent research has revealed that α-glucan exhibits good biocompatibility and biodegradability as a vaccine adjuvant. Additionally, they can maintain a stable intestinal environment within the body.^87,88^ In addition, α-(1→4)-GalpA and α-(1→4)-Gal in the polysaccharide backbone of ginseng are crucial for its antitumor properties.^89^ The main types of glycosidic bonds in active polysaccharides vary depending on their type. Glucans with antitumor properties primarily consist of β-(1→3)-d-glucan as the main chain and β-(1→6)-d-glucan as a branch chain. In contrast, glucans with β-(1→6)-d-glucan as the main chain exhibit significantly weaker antitumor effects.^90^ Polysaccharides that regulate intestinal floral activity are primarily linked *via* (1→3) glycosidic bonds.^91^ Most medicinal plant polysaccharides with hypoglycemic effects consist of (1→3), (1→4), and (1→6) glycosidic bonds.^92–94^ To optimize the use of natural sugar products, strengthening the fundamental research on carbohydrate component biology is imperative. Further research on the development and application of natural polysaccharides will enhance our understanding of their mechanisms of action and the relationship between their pharmacological functions and structures. Analysis of the structure of carbohydrate components in medicinal plants has paved the way for future research, development, and application of polysaccharides in TCM as functional foods and therapeutic agents in modern medicine. Additionally, this study provides technical support for quality control of carbohydrate components in medicinal plants. Ensuring public safety and promoting the development of the Chinese medicine industry are highly important.

This paper provides a comprehensive review of research progress in the extraction, purification, separation, analysis, and content determination of carbohydrate components in medicinal plants. Moreover, this review compares the advantages and disadvantages of different treatment methods and discusses the prospects of carbohydrate components in medicinal plants. More focus should be given to research methods and the correlation between the saccharide structure and efficacy. This will contribute to the scientific reference for the detection, analysis, development, and application of different carbohydrate components in medicinal plants, and serve as technical guidance for the future development and application of natural carbohydrate components in functional food and medicine.

## Data availability

No data was used for the research described in the article.

## Author contributions

Chao Ji: formal analysis, investigation, methodology, validation, writing – original draft, writing – review & editing. Ying Ma: methodology, validation, writing – original draft, writing – review & editing. Yuxin Xie: investigation, visualization, writing – original draft. Junli Guo: investigation, visualization. Haoran Ba: methodology, validation, formal analysis. Zheng Zhou: methodology, validation. Kongxiang Zhao: supervision, writing – review & editing. Min Yang: funding acquisition, supervision, writing – original draft. Xiahong He: funding acquisition, supervision, writing – review & editing. Wenjie Zheng: conceptualization, funding acquisition, writing – review & editing.

## Conflicts of interest

There are no conflicts to declare.
